# Diurnal variation in blood‐based biomarkers for neurodegeneration and the circadian markers melatonin and cortisol in Alzheimer's

**DOI:** 10.1002/alz.089201

**Published:** 2025-01-09

**Authors:** Ciro della Monica, Victoria Louise Revell, Giuseppe Atzori, Rhiannon Laban, Simon S. Skene, Amanda J Heslegrave, Hana Hassanin, Ramin Nilforooshan, Henrik Zetterberg, Derk‐Jan Dijk

**Affiliations:** ^1^ UK Dementia Research Institute, Care Research and Technology Centre, Imperial College London and the University of Surrey, Guildford UK; ^2^ Surrey Sleep Research Centre (SSRC), University of Surrey, Guildford, Surrey UK; ^3^ UK Dementia Research Institute at UCL, London UK; ^4^ Surrey Clinical Trial Unit, Guildford UK; ^5^ UCL Queen Square Institute of Neurology, London UK; ^6^ Surrey Clinical Research Facility, University of Surrey, Guildford, Surrey UK; ^7^ Surrey and Borders Partnership NHS Foundation Trust, Chertsey UK; ^8^ UK Dementia Research Institute, Care Research and Technology Centre, London UK; ^9^ Hong Kong Center for Neurodegenerative Diseases, Hong Kong China; ^10^ Department of Neurodegenerative Disease, UCL Queen Square Institute of Neurology, University College London, London, ‐ UK; ^11^ Wisconsin Alzheimer's Disease Research Center, University of Wisconsin School of Medicine and Public Health, Madison, WI USA; ^12^ UK Dementia Research Institute, University College London, London UK; ^13^ Department of Psychiatry and Neurochemistry, Institute of Neuroscience and Physiology, the Sahlgrenska Academy at the University of Gothenburg, Mölndal Sweden; ^14^ Department of Psychiatry and Neurochemistry, Institute of Neuroscience and Physiology, The Sahlgrenska Academy at the University of Gothenburg, Mölndal Sweden

## Abstract

**Background:**

Disruption in diurnal rest‐activity rhythms is a hallmark of Alzheimer’s disease. Currently, we know little about how physiology, symptoms, and biomarkers change over the 24‐hour day in people living with Alzheimer’s disease. In particular, we don’t know whether plasma biomarkers of neurodegeneration, which offer promise as diagnostic or stratification tools, vary with time of day, and whether these associate with the circadian markers melatonin and cortisol.

**Methods:**

We studied 38 participants (70.8 ± 7.6 years; mean ± SD) in a 27‐hour laboratory protocol including a sleep episode with either two blood samples taken 12 hours apart (n = 17) or 3‐hourly blood sampling for 24 hours (n = 21). The participants were people living with mild Alzheimer’s disease (PLWA, n = 8), their partners/caregivers (n = 6), and cognitively intact older adults (n = 24). The neurodegeneration biomarkers phosphorylated tau (p‐tau217), amyloid‐beta 40 (Aβ40), amyloid‐beta 42 (Aβ42), glial fibrillary acidic protein (GFAP), and neurofilament light (NfL) levels were measured with single molecule array technology. A mixed linear model (PROC MIXED, SAS) was run to assess the impact of time‐of‐day, group, and any interaction. The gold‐standard markers of the circadian clock, plasma melatonin and cortisol, were measured using liquid chromatography mass spectrometry (LC‐MS) for the 24‐hour timeseries (n = 21). Correlations were run (R) to explore relationships between 24‐hour levels of dementia biomarkers, melatonin and cortisol.

**Results:**

Diurnal rhythms in melatonin and cortisol were observed in all participant groups. Significant time of day variation was observed for p‐tau217, Aβ40, Aβ42, NfL, and a significant effect of group for p‐tau217 with highest levels in PLWA. There were no significant correlations between 24‐hour levels of melatonin or cortisol and any of the neurodegeneration biomarkers.

**Conclusions:**

Time of day variation was observed in central circadian hormone markers and commonly used biomarkers of dementia in all participants. Outputs of the central circadian clock were not markedly altered in PLWA, and there was no relationship between 24‐hour levels of these outputs and blood‐based biomarkers of dementia. Time of day variation in biomarkers may be relevant to their implementation and interpretation in research and clinical practice.

